# Diagnostic delay and phenotypic differences in cardiac sarcoidosis: a descriptive study of diagnostic and follow-up clinical data

**DOI:** 10.1136/openhrt-2025-003934

**Published:** 2026-02-24

**Authors:** Susanna Kullberg, Jonas Faxén, Julia Cagan, Hasti Torabzadeh, Anders Eklund, Anna Smed-Sörensen, Pernilla Darlington, Per Eldhagen, Marios Rossides

**Affiliations:** 1 Division of Immunology and Respiratory Medicine, Department of Medicine Solna, Karolinska Institutet, Center for Molecular Medicine, Karolinska University Hospital, Stockholm, Sweden; 2 Department of Respiratory Medicine and Allergy, Theme Inflammation and Ageing, Karolinska University Hospital, Stockholm, Sweden; 3 Unit of Cardiology, Theme Cardiovascular and Neurology, Karolinska University Hospital, Stockholm, Sweden; 4 Department of Physiology and Pharmacology, Karolinska Institutet, Stockholm, Sweden; 5 Department of Internal Medicine, Södersjukhuset, Stockholm, Sweden; 6 Department of Clinical Science and Education, Södersjukhuset and Karolinska Institutet, Södersjukhuset and Karolinska Institutet, Stockholm, Sweden; 7 Unit of Cardiology, Department of Medicine Solna, Karolinska Institutet, Stockholm, Sweden; 8 Unit of Epidemiology, Institute of Environmental Medicine, Karolinska Institutet, Stockholm, Sweden, Sweden

**Keywords:** Outcome Assessment, Health Care, Rare Diseases, Cardiomyopathies

## Abstract

**Background:**

Worse prognosis in cardiac sarcoidosis (CS) is likely associated with diagnostic delay and cardiac involvement as first sarcoidosis (de novo) presentation, but data are limited.

**Methods:**

We retrospectively investigated 95 patients with CS diagnosed 2003–2024. Using electronic health records, the date of first CS symptoms/signs, immunosuppressant therapy and follow-up data including left ventricular ejection fraction (LVEF), biomarkers and cardiac device therapy were extracted. Median time from first symptoms/signs to CS diagnosis (9 months) was used to define delayed diagnosis.

**Results:**

Implantation of cardiac resynchronisation therapy defibrillator was more likely in patients with diagnostic delay (p=0.01). No difference was observed in time to diagnosis between patients with de novo CS (n=49) and those with prior extracardiac sarcoidosis (ECS) (n=46). Severe symptoms at disease onset were more common in de novo CS. At a median of 46 months from diagnosis, de novo patients more often had reduced LVEF (p=0.006) and an implantable cardioverter defibrillator (p<0.05) than those with prior ECS despite receiving more immunosuppressant therapy. De novo patients with diagnostic delay more often had reduced LVEF at CS presentation.

**Conclusions:**

Symptom presentation is likely associated with diagnostic delay, but the disease presentation and course seem more severe in de novo CS and may not be altered by immunosuppressants, or demand more aggressive therapy.

**WHAT IS ALREADY KNOWN ON THIS TOPIC**

- Worse prognosis in cardiac sarcoidosis is associated with diagnostic delay and presenting phenotype, but their relative contribution and the influence of immunosuppressive therapy are unclear.

**WHAT THIS STUDY ADDS**

- Cardiac sarcoidosis phenotype, that is, cardiac manifestation as first presentation versus cardiac sarcoidosis presenting after extra cardiac sarcoidosis, may be more important than diagnostic delays for disease presentation and disease course. The phenotypic differences do not seem to be reversed by conventional immunosuppressive treatment.

**HOW THIS STUDY MIGHT AFFECT RESEARCH, PRACTICE OR POLICY**

- Early detection is important, but phenotype is equally important for disease presentation and course, indicating a need for phenotype-based risk stratification in the clinic and future research.

## Introduction

 Sarcoidosis is a systemic inflammatory disease, affecting the lungs and intrathoracic lymph nodes in most cases, but the disease can occur in almost any organ.^1^ After pulmonary sarcoidosis, cardiac sarcoidosis (CS) is a leading cause of death among affected patients and often presents as high-degree atrioventricular block (AVB), ventricular arrhythmia or heart failure (HF).^2^,^4^ Patients with CS as first sarcoidosis presentation (de novo) usually have limited extracardiac sarcoidosis (ECS) manifestations, a more severe disease and worse outcome than patients already diagnosed with ECS.^4^,^10^ As no specific test is available to diagnose CS and the clinical presentation often mimics other conditions, diagnosis can be delayed.^8 11^ Few studies have addressed the impact of diagnostic delay on symptom presentation, disease course and outcomes. The results have been conflicting, though the majority have shown an association between worse disease course, outcome and diagnostic delay.^12^,^16^

Current CS treatment algorithms focus on immunosuppressive therapy, that is, oral corticosteroids, cytotoxic agents and tumour necrosis factor α inhibitors (TNFi) as first, second and third line options, respectively. They also highlight the role of implantable cardiac devices, such as pacemakers (PM), implantable cardioverter defibrillators (ICD) or cardiac resynchronisation therapy defibrillator (CRT-D) and pharmacological therapies to manage arrhythmias and treat left ventricular dysfunction. However, whether immunosuppressive therapy may alter disease course or improve outcomes is unknown.^2 4 17^ Some data suggest that therapy has been associated with recovery in patients with AVB, but results on the effect from immunosuppressants on left ventricular function, the key survival indicator, are conflicting.^17^

Taken together, it is unclear whether the more severe symptom presentation, disease course and worse outcomes observed in patients with de novo CS are due to diagnostic delays or whether this phenotype is associated with more severe disease, or differences in immunosuppressive treatment patterns. To disentangle the possible contribution of diagnostic delays and phenotype (CS de novo vs already diagnosed ECS) to symptom presentation and disease course, we studied cardiac biomarkers and left ventricular ejection fraction (LVEF) at disease presentation and follow-up, taking treatment including implantable cardiac devices into account.

## Methods

### Study subjects

We initially considered 108 consecutive patients diagnosed with CS at the Departments of Respiratory Medicine and Cardiology, Karolinska University Hospital in Stockholm between 2003 and 2024. Medical records were reviewed by four of the authors (SK, JC, HT and MR). Patients who did not fulfil Japanese Circulation Society (JCS) or Heart Rhythm Society (HRS) criteria for CS^18 19^ (n=4), and patients who later received another diagnosis or were initially diagnosed at another hospital whose records were not available (n=9) were excluded.

### Phenotypic characterisation

We performed a form-based standardised extraction of data from patients’ electronic health records. Demographic data included birth date and sex (female/male). Clinical data included the date of first symptoms related to CS (eg, symptoms suggestive of HF or arrhythmia) and date of first signs compatible with CS (eg, ECG or echocardiographic abnormalities). Patients were divided into two groups depending on when CS appeared: (1) de novo CS defined as CS symptoms/signs presenting before ECS diagnosis and any ECS symptoms/signs and (2) CS with prior ECS defined as CS presenting simultaneously or after symptoms/signs of ECS. Subsequently, in the de novo group, both patients with isolated CS and patients with ECS not causing symptoms were included. In the prior ECS group, patients with previously diagnosed ECS were included as well as patients with symptoms of CS and ECS presenting simultaneously, for instance, patients presenting with cough due to lung parenchymal involvement and high-degree AVB due to CS.

We further evaluated information on ECG, implantation of cardiac device (PM, ICD or CRT-D), plasma N-terminal-prohormone of brain natriuretic peptide (p-NT-proBNP), p-Troponin T, serum angiotensin converting enzyme (s-ACE) levels, LVEF estimated with echocardiography, chest X-ray and lung function parameters around the time of diagnosis before initiation of immunosuppressive treatment. Chest X-rays were staged according to Scadding (0=no visible intrathoracic pathology compatible with sarcoidosis, I=lymphadenopathy, II=parenchymal infiltrates and lymphadenopathy, III=parenchymal infiltrates, IV=lung fibrosis).^20^ Date of CS diagnosis was defined as the earliest date when the diagnostic criteria according to HRS or JCS were met and stated by a treating physician in free text in the patient’s health record as no specific ICD codes are available for CS.

Follow-up data included vital status, presence of cardiac device therapy and the most recent assessment of LVEF, p-NT-proBNP and/or p-Troponin T. The latter were not always performed or measured simultaneously; therefore, they were assessed at different follow-up times. Reference values for p-NT-proBNP, p-Troponin T and s-ACE changed during the study period. To account for those changes, we dichotomised values using the period-specific upper limit of normal as cut-off, with values above the cut-off considered as elevated. Patients on treatment with ACE inhibitors were excluded from analysis of s-ACE.

Treatments were defined as all previous and current immunosuppressive therapies including oral corticosteroids, cytotoxic agents (mostly methotrexate, a few with azathioprine), and TNFi administered up to the date of follow-up with echocardiography and biomarker measurement. In our cohort, treatment was initiated adjacent to the date for CS diagnosis. Patients were usually started on 30 mg prednisone per day, tapered to 20 mg during the first 2 months, followed by 15 mg per day for 3 months, 10 mg per day for 6 months and finally tapered out within 2 months. If patients showed signs of increased disease activity (eg, deterioration of left ventricular function, arrhythmia, increasing p-NT-proBNP and/or p-Troponin T) during dose reduction, ^18^F-FDG-PET was usually performed to verify the relapse. If relapse occurred at a prednisone dose exceeding 10 mg per day, methotrexate was usually added (7.5 mg per week, which was gradually increased to 15 mg weekly within 2 months). Otherwise, prednisone dose was increased to the lowest maintenance dose possible. Patients not responding to first or second-line therapies received TNFi.

### Statistical analysis

We estimated the median time from first symptoms or signs (whichever came first) to CS diagnosis in the whole study population (ie, 9 months) and used it as cut-off to define delayed diagnosis (≥9 months).

We then performed several comparative analyses. First, median time from first symptoms/signs to CS diagnosis was compared by age, sex, timing of CS diagnosis (de novo CS vs prior ECS) and other clinical variables described above. Second, we compared patient characteristics among those with and without delayed CS diagnosis (≥9 months vs <9 months from first symptoms/signs, respectively). In addition, analyses were repeated by stratifying into three groups considering time to CS diagnosis (0–2.9, 3.0–8.9 and ≥9 months) to identify likely indicators of early and very early CS diagnosis, and by phenotype (de novo CS and CS with prior ECS). Finally, patients with de novo CS and those with prior ECS were further stratified by diagnostic delay.

Groups in all analyses were compared using Mann-Whitney U or Kruskal-Wallis tests for continuous variables, or χ^2^ or Fisher’s exact tests, as appropriate, for categorical variables. We considered p values from two-tailed tests <0.05, indicating statistical significance. Missing values were marked as such and analysed as a separate category; no patients were excluded from analyses based on missing values.

### Patient and public involvement

Patients or the public were not involved in the design, conduct, reporting or dissemination plans of our research.

## Results

### Patient characteristics and time to CS diagnosis

Ninety-five patients were included in the final analysis and about half of these were also included in two previous publications.^5 6^ All patients fulfilled the JCS criteria and 73 (77%) also met the HRS criteria. Nine patients had isolated CS, three of these fulfilled the HRS and six only the JCS criteria. Out of the 73 patients that met the HRS criteria, 9 had a positive endomyocardial biopsy and 66 a positive extracardiac biopsy. During the diagnostic work-up, 57 patients (60%) underwent ^18^F-fluorodeoxyglucose positron emission tomography (^18^F-FDG-PET), and 80 (84.2%) cardiac MRI (CMRI). Two patients had both a positive endomyocardial and extracardiac biopsy and some were investigated with both ^18^F-FDG-PET and CMRI.

**Figure 1:**
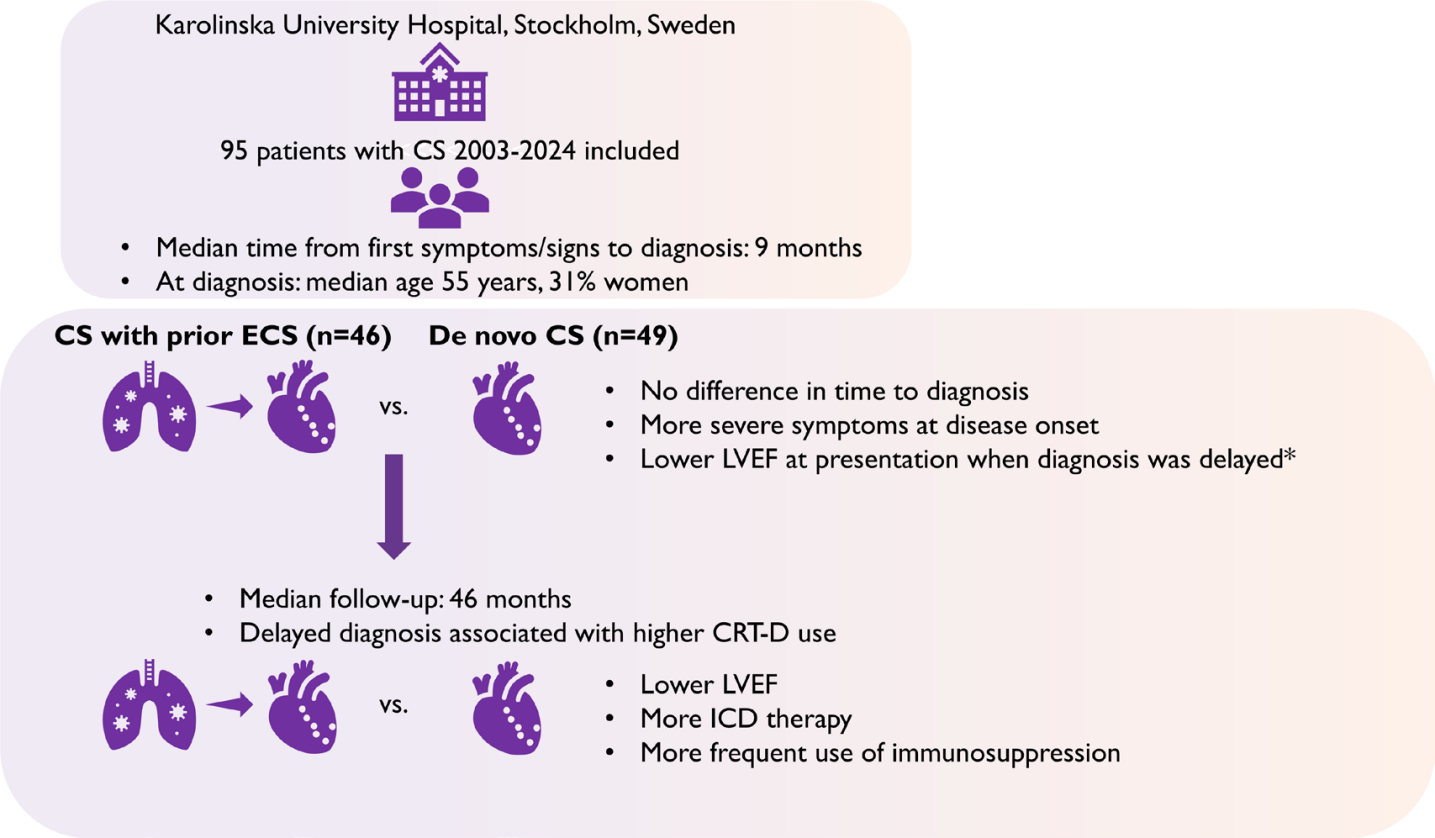
Depiction of the studies’ main findings. Delayed diagnosis was defined as ≥9 months from first symptoms or signs of CS to diagnosis. CRT-D, cardiac resynchronisation therapy defibrillator; CS, cardiac sarcoidosis; ECS, extracardiac sarcoidosis; ICD, implantable cardioverter defibrillator; LVEF, left ventricular ejection fraction.

Demographic and clinical characteristics at diagnosis are shown in table 1 and figure 1. The median age at CS diagnosis was 54.8 years (IQR 46.7–61.4); 29 patients (31%) were women. CS diagnosis was established within a median of 9.3 months (IQR 3.1–20.2) from first symptoms/signs attributable to CS and did not differ by phenotype, that is, de novo CS versus simultaneously or after ECS diagnosis (table 1).

**Table 1:** Median months from first symptoms or signs to CS diagnosis by patient characteristics

	N (%)	Median months from first symptoms or signs to CS diagnosis (IQR)	P value
N patients	95	9.3 (3.1–20.2)	
Age, years			
<55	48 (50.5)	5.5 (2.0–15.0)	0.001
≥55	47 (49.4)	15.0 (5.0–40.0)	
Sex			0.58
Female	29 (30.5)	9.0 (3.0–15.0)	
Male	66 (69.5)	10 (3.0–20.0)	
Timing of CS diagnosis			0.69
Before ECS	49 (51.6)	10.0 (4.0–20.0)	
Simultaneously	19 (20.0)	5.0 (3.0–18.0)	
After ECS	27 (28.4)	12.0 (2.0–25.0)	
Calendar period			0.33
≤2015	29 (30.5)	8.0 (3.0–15.0)	
≥2016	66 (69.5)	10.0 (3.0–24.0)	
LVEF, %			0.13
≥50	50 (52.6)	7.5 (2.0–17.0)	
<50	35 (36.8)	15.0 (3.5–36.0)	
Missing	10 (10.5)	8.5 (4.0–11.0)	
Heart failure symptoms			0.16
Yes	27 (28.4)	14.0 (4.0–36.0)	
No	68 (71.6)	8.0 (3.0–19.0)	
Arrhythmia symptoms			0.74
Yes	56 (59.0)	11.5 (3.0–19.5)	
No	39 (41.1)	8.0 (3.0–24.0)	
High-grade AVB			0.23
Yes	27 (28.4)	9.0 (3.0–18.0)	
No	67 (70.5)	10.0 (3.0–24.0)	
Missing	1 (1.1)	0.0 (0.0–0.0)	
Ventricular tachycardia			0.25
Yes	30 (31.6)	13.5 (4.0–36.0)	
No	65 (68.4)	8.0 (3.0–18.0)	
FVC<80% predicted			0.85
Yes	34 (35.8)	9.5 (3.0–30.0)	
No	46 (48.4)	8.5 (3.0–17.0)	
Missing	15 (15.8)	12.0 (1.0–27.0)	
Scadding stage			0.21
0	34 (35.8)	8.0 (4.0–18.0)	
I	11 (11.6)	8.0 (2.0–10.0)	
II	36 (37.9)	15.0 (2.5–24.5)	
III	7 (7.4)	12.0 (2.0–14.0)	
IV	6 (6.3)	27.0 (9.0–129.0)	
Missing	1 (1.1)	0.0 (0.0–0.0)	
S-ACE			0.81
Elevated	23 (24.2)	12.0 (3.0–24.0)	
Normal	68 (71.6)	8.5 (3.0–22.0)	
Missing	4 (4.2)	9.5 (4.0–14.5)	
Died			0.65
Yes	7 (7.3)	18.0 (5.0–24.0)	
No	88 (92.6)	9.0 (3.0–20.0)	

Data are presented as N (%) or median (IQR).

AVB, atrioventricular block; CS, cardiac sarcoidosis; ECS, extracardiac sarcoidosis; Calendar period refers to year of CS diagnosis; FVC, forced vital capacity; LVEF, left ventricular ejection fraction from echocardiography; S-ACE, serum angiotensin converting enzyme; Scadding, radiographic extent of sarcoidosis assessed by chest X-ray using Scadding staging system (0–IV).

The median time to CS diagnosis was 15.0 months (IQR 5.0–40.0) in individuals ≥55 years, while younger patients were diagnosed at a median time of 5.5 months from first signs/symptoms (IQR 2.0–15.0) (p=0.001; table 1). Patients ≥55 years at CS diagnosis were more likely to have elevated p-NT-proBNP and p-Troponin T at follow-up (p=0.02 and <0.001, respectively) than those younger, data not shown. All patients who died until follow-up (seven patients by 31 March 2025) belonged to the older age group (p=0.006).

As shown in table 2, those with a diagnostic delay ≥9 months were more likely to receive a CRT-D (p=0.01) and present with HF symptoms (p=0.07). Other parameters did not differ between those with and without diagnostic delay. Similar results were obtained when patients were divided into three groups depending on time to CS diagnosis (0–2.9, 3.0–8.9 and ≥9 months); 4.4% of patients in the early and very early group had CRT-D compared with 22.5% in the diagnostic delay group (p=0.04), data not shown.

**Table 2:** Characteristics of patients with diagnostic delay defined as ≥9 months from first symptoms or signs of CS to diagnosis

	Diagnostic delay		P value
	Yes	No	
N patients	49	46	
At CS diagnosis			
Timing of CS diagnosis			0.48
De novo CS	27 (55.1)	22 (47.8)	
Prior ECS	22 (44.9)	24 (52.2)	
LVEF, %			0.10
≥50	21 (42.9)	29 (63.0)	
<50	23 (46.9)	12 (26.1)	
Missing	5 (10.2)	5 (10.9)	
Heart failure symptoms	18 (36.7)	9 (19.6)	0.07
Arrhythmia symptoms	30 (61.2)	26 (56.5)	0.64
High-grade AVB			0.91
Yes	14 (28.6)	13 (28.3)	
No	35 (71.4)	32 (69.6)	
Missing	0 (0.0)	1 (2.2)	
Ventricular tachycardia	17 (34.7)	13 (28.3)	0.50
Elevated p-Troponin T			0.51
Yes	28 (57.1)	28 (60.9)	
No	9 (18.4)	11 (23.9)	
Missing	12 (24.5)	7 (15.2)	
Elevated p-NT-proBNP			0.45
Yes	33 (67.4)	26 (56.5)	
No	10 (20.4)	10 (21.7)	
Missing	6 (12.2)	10 (21.7)	
At CS follow-up			
Months since diagnosis at ascertainment, median (IQR)			
Echocardiography	37.6 (19.7–77.2)	51.9 (30.1–85.5)	0.16
p-Troponin T	38.7 (26.5–61.6)	49.5 (29.0–78.2)	0.18
p-NT-proBNP	40.8 (28.7–77.3)	54.0 (29.0–85.8)	0.28
LVEF, %			0.42
≥50	25 (51.0)	20 (43.5)	
<50	19 (38.8)	17 (37.0)	
Missing	5 (10.2)	9 (19.6)	
Elevated p-Troponin T			1.00
Yes	11 (22.5)	11 (23.9)	
No	32 (65.3)	30 (65.2)	
Missing	6 (12.2)	5 (10.9)	
Elevated p-NT-proBNP			0.21
Yes	31 (63.3)	23 (50.0)	
No	15 (30.6)	22 (47.8)	
Missing	3 (6.1)	1 (2.2)	
Treatment at echocardiography			0.58
Corticosteroids only	24 (49.0)	19 (41.3)	
Corticosteroids and cytotoxic agents	18 (36.7)	17 (37.0)	
Other/none	2 (4.1)	1 (2.2)	
Missing	5 (10.2)	9 (19.6)	
Treatment at biomarker assessment			0.70
Corticosteroids only	25 (51.0)	22 (47.8)	
Corticosteroids and cytotoxic agents	19 (38.8)	22 (47.8)	
Other/none	2 (4.1)	1 (2.2)	
Missing	3 (6.1)	1 (2.2)	
Pacemaker			0.59
Yes	15 (30.6)	16 (34.8)	
No	34 (69.4)	29 (63.0)	
Missing	0 (0.0)	1 (2.2)	
ICD			0.10
Yes	22 (44.9)	28 (60.9)	
No	27 (55.1)	17 (37.0)	
Missing	0 (0.0)	1 (2.2)	
CRT-D			0.01
Yes	11 (22.5)	2 (4.4)	
No	38 (77.6)	43 (93.5)	
Missing	0 (0.0)	1 (2.2)	
Died	4 (8.2)	3 (6.5)	1.00

Data are presented as N (%) unless otherwise stated.

AVB, atrioventricular block; CRT-D, cardiac resynchronisation therapy defibrillator; CS, cardiac sarcoidosis; ECS, extracardiac sarcoidosis; ICD, implantable cardioverter defibrillator; LVEF, left ventricular ejection fraction from echocardiography; NT-proBNP, N-terminal prohormone of brain natriuretic peptide.

### Disease presentation in de novo CS versus prior ECS groups

For 49 out of 95 patients (52%), CS was the first sarcoidosis manifestation (de novo). These patients presented more often with HF symptoms (p<0.001), ventricular tachycardia (VT) (p=0.01) and LVEF lower than 50% (p=0.01) than patients with prior ECS, that is, patients with CS presenting after (n=27) or simultaneously (n=19) (table 3 and figure 1). Patients with de novo CS more often had a normal s-ACE, lower Scadding stages (p=0.002 and <0.001, respectively), and were younger than those with prior ECS (p=0.06; table 3).

**Table 3:** Characteristics of patients by timing of CS diagnosis in relation to ECS diagnosis

	All patients	Timing of CS diagnosis		P-value
		De novo CS	Prior ECS	
N patients	95	49	46	
At CS diagnosis				
Age, years; median (IQR)	54.8 (46.7, 61.4)	53.7 (45.8, 57.7)	55.1 (46.9, 66.7)	0.18
Sex				0.67
Female	29 (30.5)	14 (28.6)	15 (32.6)	
Male	66 (69.5)	35 (71.4)	31 (67.4)	
LVEF, %				0.01
≥50	50 (52.6)	20 (40.8)	30 (65.2)	
<50	35 (36.8)	25 (51.0)	10 (21.7)	
Missing	10 (10.5)	4 (8.2)	6 (13.0)	
Heart failure symptoms	27 (28.4)	23 (46.9)	4 (8.7)	<0.001
Arrhythmia symptoms	56 (59.0)	31 (63.3)	25 (54.4)	0.38
High-grade AVB				0.37
Yes	27 (28.4)	16 (32.7)	11 (23.9)	
No	67 (70.5)	32 (65.3)	35 (76.1)	
Missing	1 (1.1)	1 (2.0)	0 (0.0)	
Ventricular tachycardia	30 (31.6)	21 (42.9)	9 (19.6)	0.01
Scadding stage				<0.001
0	34 (35.8)	28 (57.1)	6 (13.0)	
I	11 (11.6)	7 (14.3)	4 (8.7)	
II	36 (37.9)	9 (18.4)	27 (58.7)	
III	7 (7.4)	4 (8.2)	3 (6.5)	
IV	6 (6.3)	0 (0.0)	6 (13.0)	
Missing	1 (1.1)	1 (2.0)	0 (0.0)	
FVC<80% predicted				0.18
Yes	34 (35.8)	16 (32.7)	18 (39.1)	
No	46 (48.4)	22 (44.9)	24 (52.2)	
Missing	15 (15.8)	11 (22.5)	4 (8.7)	
S-ACE				0.002
Elevated	23 (24.2)	5 (10.2)	18 (39.1)	
Normal	68 (71.6)	41 (83.7)	27 (58.7)	
Missing	4 (4.2)	3 (6.1)	1 (2.2)	
Elevated p-Troponin T				0.003
Yes	56 (59.0)	37 (75.5)	19 (41.3)	
No	20 (21.1)	7 (14.3)	13 (28.3)	
Missing	19 (20.0)	5 (10.2)	14 (30.4)	
Elevated p-NT-proBNP				0.15
Yes	59 (62.1)	35 (71.4)	24 (52.2)	
No	20 (21.1)	8 (16.3)	12 (26.1)	
Missing	16 (16.8)	6 (12.3)	10 (21.7)	
Diagnostic delay	49 (51.6)	27 (55.1)	22 (47.8)	0.48
At CS follow-up				
Months since diagnosis at follow-up, median (IQR)				
Echocardiography	40.1 (25.9, 79.3)	36.1 (19.7, 63.1)	72.6 (30.2, 104.0)	0.02
p-Troponin T	43.6 (27.5, 75.9)	41.9 (29.4, 67.1)	48.7 (24.2, 83.3)	0.73
p-NT-proBNP	46.4 (28.7, 80.6)	43.6 (29.9, 63.1)	56.1 (25.4, 108.4)	0.38
LVEF, %				0.006
≥50	45 (47.4)	21 (42.9)	24 (52.2)	
<50	36 (37.9)	25 (51.0)	11 (23.9)	
Missing	14 (14.7)	3 (6.1)	11 (23.9)	
Elevated p-Troponin T				0.55
Yes	22 (23.2)	11 (22.5)	11 (23.9)	
No	62 (65.3)	34 (69.4)	28 (60.9)	
Missing	11 (11.6)	4 (8.2)	7 (15.2)	
Elevated p-NT-proBNP				0.09
Yes	54 (56.8)	31 (63.3)	23 (50.0)	
No	37 (39.0)	18 (36.7)	19 (41.3)	
Missing	4 (4.2)	0 (0.0)	4 (8.7)	
Treatment at echocardiography				0.01
Corticosteroids only	43 (45.3)	20 (40.8)	23 (50.0)	
Corticosteroids and cytotoxic agents	35 (36.8)	24 (49.0)	11 (23.9)	
Other/none	3 (3.2)	2 (4.1)	1 (2.2)	
Missing	14 (14.7)	3 (6.1)	11 (23.9)	
Treatment at biomarker assessment				0.04
Corticosteroids only	41 (43.2)	21 (42.9)	26 (56.5)	
Corticosteroids and cytotoxic agents	40 (42.1)	26 (53.1)	15 (32.6)	
Other/none	2 (2.1)	2 (4.1)	1 (2.2)	
Missing	12 (12.6)	0 (0.0)	4 (8.7)	
Pacemaker				0.74
Yes	31 (32.6)	17 (34.7)	14 (30.4)	
No	63 (66.3)	32 (65.3)	31 (67.4)	
Missing	1 (1.1)	0 (0.0)	1 (2.2)	
ICD				<0.05
Yes	50 (52.6)	31 (63.3)	19 (41.3)	
No	44 (46.3)	18 (36.7)	26 (56.5)	
Missing	1 (1.1)	0 (0.0)	1 (2.2)	
CRT-D				0.56
Yes	13 (13.7)	8 (16.3)	5 (10.9)	
No	81 (85.3)	41 (83.7)	40 (87.0)	
Missing	1 (1.1)	0 (0.0)	1 (2.2)	
Died	7 (7.3)	1 (2.0)	6 (13.0)	0.05

Data are presented as N (%) unless otherwise stated.

AVB, high degree atrioventricular block; CRT-D, cardiac resynchronisation therapy defibrillator; CS, cardiac sarcoidosis; ECS, extracardiac sarcoidosis; FVC, forced vital capacity; HF, heart failure; ICD, implantable cardioverter defibrillator; LVEF, left ventricular ejection fraction from echocardiography; NT-proBNP, N-terminal prohormone of brain natriuretic peptide; S-ACE, serum angiotensin converting enzyme; Scadding, radiographic extent of sarcoidosis assessed by chest X-ray using Scadding staging system (0–IV).

### Follow-up data and treatment in de novo CS versus prior ECS groups and the role of diagnostic delay

There were no differences in follow-up times between de novo CS and prior ECS patients, except for echocardiography, which was performed at a median of 36 months (IQR 19–63) and 73 months (IQR 30–104) in de novo CS and prior ECS patients, respectively (p=0.01), shown in table 3. Compared with patients with prior ECS, those with de novo CS were more likely to have reduced LVEF <50% (p=0.006) and have received an ICD (p<0.05) at follow-up (table 3, figure 1). There were more patients with de novo CS who had been treated with corticosteroids and cytotoxic agents than corticosteroids alone compared with patients with prior ECS, both at follow-up echocardiography and at biomarker follow-up (p=0.01 and 0.04, respectively; table 3). TNFi treatment could not be analysed separately due to small numbers.

When stratifying patients by time from first symptoms or signs to CS diagnosis, 27 out of 49 (55%) in the de novo group and 22 out of 46 (48%) in the prior ECS group received their diagnosis ≥9 months after presenting first CS symptoms/signs. Patients with de novo CS with diagnostic delay presented more often with lower LVEF (p=0.04) and were more likely to receive a CRT-D (p=0.06) compared with those who received their diagnosis earlier (data not shown). In the prior ECS group, no differences were observed except for CRT-D, where only one patient diagnosed <9 months received that therapy (4.2%) compared with four patients in the delayed group (18.2%).

## Discussion

In this study, we sought to understand the possible contribution of diagnostic delays and phenotype (de novo CS vs already diagnosed ECS) for symptom presentation and disease course, evaluated with biomarkers and LVEF, in a Swedish cohort of CS patients, taking immunosuppressive treatment into account. It has been hypothesised that the reason for de novo CS having a more severe disease presentation and worse disease course than CS with prior ECS is due to less or lack of ECS, anticipating that diagnosis could be missed and/or difficult to establish.^5 6 8 15^ Also, in this study, patients with de novo CS showed less ECS than patients with prior ECS, evidenced by lower s-ACE and earlier Scadding stage. But even though the median time from first CS symptoms/signs did not differ between the two phenotypes, patients with de novo CS presented more often with HF symptoms, VT and lower LVEF than patients with prior ECS. Also, at follow-up, de novo CS patients showed signs of worse disease course—they had more often reduced LVEF and an ICD and had received second-line treatment more frequently. However, as diagnostic delay was associated with CRT-D implantation, it seems to influence disease severity to some extent, which is in line with previous reports linking diagnostic delay and late initiation of treatment to more severe CS symptoms and outcome.^12^,^1416 21 22^ Interestingly, when comparing patients with de novo CS and prior ECS with and without diagnostic delay, the only significant differences we observed were that de novo CS patients with diagnostic delay more often had reduced LVEF at disease presentation and were more likely to receive a CRT-D at follow-up than de novo CS patients without diagnostic delay. There were no statistically significant differences in LVEF between those with and without diagnostic delay in the prior ECS group, although a greater number of patients with diagnostic delay received a CRT-D at follow-up. Therefore, disease presentation and course appear to be influenced by diagnostic delay, especially in de novo CS. However, the association can also go the other way round; symptom presentation precedes the diagnosis of CS and may thus influence diagnostic delay.

Surprisingly, we did not detect larger differences in follow-up data between those with and without diagnostic delay as previous studies have shown more clear differences,^12^,^14^ and the median time to diagnosis in our cohort was longer than what is reported from other cohorts.^12 16 23^ However, results should be compared with caution as inclusion criteria and outcome measurements differed considerably among studies. The lack of more robust differences between those with and without diagnostic delay could be explained by the fact that the definition of diagnostic delay used in this study did not demand objective signs of CS but also considered patients’ first reported symptoms. In contrast, previous studies defined diagnostic delay by objective observations.^12^,^1416^ Having used the same objective criteria in our study, the time to diagnosis would have been shorter. There is no consensus regarding the appropriate cut-off for delayed diagnosis of CS. Both median time to diagnosis and arbitrary definitions have been used, further hindering comparisons.^12^,^15^ It should also be noted that there are studies reporting that outcome was independent of diagnostic delays,^8 15^ but that diagnostic delay may influence disease presentation.^15^

Patients with de novo CS seemed to have a worse disease course even though they were more often treated with corticosteroids and cytotoxic agents. This is in line with results from a previous study of ours with a partially overlapping population.^5^ Although there were no differences in immunosuppressant treatment patterns between patients with de novo CS and those with prior ECS, de novo CS was associated with worse outcome assessed with a composite consisting of ventricular arrhythmias, heart transplantation and death.^5^ Also, the study by Rosen *et al*^9^ points in the same direction; patients with CS de novo had an increased risk of adverse cardiac outcomes despite being treated with corticosteroids more often than those with prior ECS. It should be noted though, in that study, the prescription of steroid-sparing agents did not differ between the groups but mycophenolate mofetil was more commonly used in the de novo CS group and the corticosteroid dose was higher in the prior ECS group, which may have partly influenced disease course. Interestingly, Fussner *et al* concluded that clinical presentation of CS has a larger impact on outcomes than treatment regimen.^24^ In their study, which included 91 patients from two large academic centres, the presence of cardiomyopathy at diagnosis was associated with worse outcome than presence of AVB or VT. This is in line with findings from other studies. In fact, current data indicate improvement in AV nodal conduction, while the effect on LVEF remains uncertain; possibly deterioration is avoided at least in those with preserved LVEF.^1725^,^28^ We observed a trend towards diagnostic delay in patients with HF symptoms at diagnosis. Thus, one explanation for the lack of improvement of LVEF from immunosuppressants may be that therapy initiation is also delayed. But it is also possible that these patients would benefit from more aggressive immunosuppressive treatment.

In our study, there were more deaths in the prior ECS group, which is in contrast to the findings of the present and previous studies indicating that de novo CS is a phenotype associated with worse symptoms and outcome.^4^,^9^ However, all deaths occurred in older patients and diagnostic delay is more common in older age. A plausible explanation for these observations may be that cardiac symptoms in older patients initially were assigned to cardiac conditions more common in an older population, and the worse disease course due to delayed treatment initiation. It may also be possible that comorbidities or other cardiac conditions more common in the elderly influence cardiac impairment. An association with age and late diagnosis was reported previously.^12^ Our findings are also in line with those recently reported by Nabeta *et al,* showing that the risk of all-cause death and HF hospitalisation was higher in older patients compared with other age groups but the older age group also had more comorbidities.^29^ As we did not investigate comorbidities in this group, we are unable to shed more light on this issue.

Strengths of this study include a well-defined, relatively large patient group, composed of mainly white ancestry individuals of Nordic descent, all well characterised phenotypically with a long observation time. All data, including the CS diagnosis, were verified by specialists in respiratory medicine and cardiology.

There are some limitations, including the retrospective approach of this study. We included a population with an almost uniform ethnic composition diagnosed at a highly specialised tertiary centre, two factors which may limit comparisons with other populations. In addition, we lacked data on all parameters for some patients resulting in missingness, which may have contributed to some differences in stratified analyses. Phenotypic stratification is a considerable strength of this study but resulted in small numbers in some groups. Furthermore, we did not study the length and dosing of either immunosuppressive or other pharmacologic therapies. However, all patients were followed at a specialist centre with similar routines for immunosuppressive and cardiac-specific treatments, but it cannot be ruled out that there are differences in treatment patterns that may have influenced the results. Finally, different follow-up times for LVEF estimation between de novo CS and prior ECS groups could explain the higher frequency of reduced LVEF in the de novo CS group. However, as LVEF was not the only parameter that differed, it is unlikely that different follow-up times are of considerable importance.

To conclude, we found that disease presentation may influence diagnostic delay. Importantly, disease presentation and course evaluated by cardiac biomarkers and LVEF vary considerably by CS phenotype and is likely more severe in de novo CS than CS with prior extracardiac disease. Phenotypic differences do not seem to be reversed by immunosuppressive treatment, at least not by the immunosuppressants administered to the patients in this study. Stratifying patients by phenotype may be considered in future research studies and for optimal clinical follow-up.

## Data Availability

Data are available upon reasonable request.
